# Towards Cleaner Production Ecosystem: An Analysis of Embodied Industrial Pollution in International Trade of China’s Processing versus Normal Exports

**DOI:** 10.3390/ijerph19169900

**Published:** 2022-08-11

**Authors:** Yuting Dang, Yating Song, Muhammad Mohiuddin, Dan Sheng

**Affiliations:** 1 School of Management, Tianjin University of Technology, Tianjin 300084, China; 2 Faculty of Business Administration, Laval University, Quebec, QC G1S2K7, Canada; 3 School of Economics, Nankai University, Tianjin 300071, China

**Keywords:** non-competitive input-output table, processing trade, embodied domestic emission in exports, the balance of embodied emission in trade, pollution terms of trade, pollution haven hypothesis

## Abstract

While promoting economic growth, industrial development is causing serious environmental problems and threatening human health. Studies on pollution transfer through international trade often over-estimate the actual embodied emissions in exports and ignore the industrial pollutants. By designing a non-competitive input-output model which differentiates between processing exports and normal exports, we calculate the embodied domestic and imported industrial emissions in China’s processing and normal exports and imports. We also calculate the balance of embodied emission in trade (*BEET*) and the pollution terms of trade (*PTT*), as well as the decomposition of scale, structural, and technical effects on embodied emission in international trade. The results demonstrate that processing exports reduce domestic pollution by importing intermediate inputs; normal exports, on the other hand, have a considerable impact on domestic pollution. Bilateral trade between China and the US has the most detrimental impact on China’s local environment, followed by trade between China and Japan. China’s exports to Japan are more polluting per unit than those to the US and Germany. Technological upgradations and transformation of trade structure have helped to reduce the negative environmental consequences of China-US and China-Japan bilateral trade. Investment in technology and trade policy can lead to a cleaner production ecosystem.

## 1. Introduction

The transfer of the embodied industrial emissions through bilateral trade between China and her top trading partners and its impact on local industrial pollution is an important issue. Sixty percent of global trade consists of intermediate components under the global value chain (GVC) framework [1]. Due to the lack of complete data on the global trade in value added (TiVA), it is challenging to identify and quantify the exact contributions of individual countries to production, as well as pollution and environmental degradation. Previous studies face criticism in the form of overestimation of embodied pollution in international trade due to the absence of a distinction between processing and normal trade. The processing trade refers to the business activities in which enterprises import all or part of the raw and auxiliary materials, parts, primary components, and packaging materials and then re-export finished products after processing. Common processing trade includes processing with imported materials, processing with supplied materials, assembly business, and collaborative production networks. Because the proportion of imports used in processing exports is much higher than that of normal exports, traditional measurements without distinguishing between processing exports and normal exports will result in an overestimation of embodied pollution in exports of countries with a high share of processing trade, such as China and Mexico. There is no comprehensive study yet of pollution transfer in bilateral trade between China and its key industrialized trading partners that distinguishes between processing exports and normal exports. 

Industrial pollution is one of the leading causes of pollution worldwide. In the United States, for example, the Environmental Protective Agency estimates that up to 50% of the nation’s pollution is caused by industry. Because of its size and scope, industrial pollution is a serious problem for the world, especially in nations that are rapidly industrializing, such as China. On the one hand, industrial development drives economic growth, promotes employment and raises people’s living standards, but on the other hand, pollutant emissions from industrial production cause serious environmental problems and threaten human health. According to [2], “Ambient (outdoor air pollution) in both cities and rural areas was estimated to cause 4.2 million premature deaths worldwide in 2016.” Industrial pollutants have deleterious effects on life expectancy and infant mortality by adversely influencing the respiratory system [3,4], nervous system [5,6,7], lungs [8,9], pregnancy outcomes [10,11], and cardiovascular system [12,13].

Since China’s accession to the World Trade Organization (WTO) in 2001, it recorded an annual export growth of 29% and became the second-largest global trader in 2010 [14,15]. Processing trade made China a “major trading and manufacturing hub” facilitated by economic reforms such as “*Made in China 2025*”. It brings benefits to global consumers by exporting low-cost, high-volume, and labor-intensive competitive products [16]. However, this production has made China gradually become a “pollution heaven” [17]. Export-oriented low-tech production has become an essential driver of air pollution in China [18] and negatively impacts the environmental ecosystem [19,20]. It threatens human health [21,22], with an estimated 12% of China’s total mortality attributable to PM_2.5_-related air pollution in 2007 [23].

By calculating the embodied emissions using an input-output model without differentiating processing trade from normal trade, several studies look at the pollution transfer in international trade between China and the USA. Those models include the competitive I-O table. Depending on the treatment of imported goods, input-output models can be divided into competitive and non-competitive input-output models. In competitive input-output models, the intermediate inputs consumed by each production sector are not differentiated between domestically produced and imported goods, and they are assumed to be fully substitutable, with only one import column vector in the final demand quadrant. Thus, this type of input-output model does not capture the link between production sectors and imported goods. The intermediate inputs in the non-competitive input-output model are divided into two major components: domestically produced intermediate inputs and imported intermediate inputs, reflecting the imperfect substitutability between the two [24,25,26], the non-competitive multi-regional I-O table (MRIO) [27,28], and China’s official non-competitive single region I-O table (SRIO) [29]. However, when embodied emissions in exports are assessed without making a distinction between normal and processing exports, the real embodied emissions in exports from countries with a high proportion of processing trade are overestimated.

**Figure 1:**
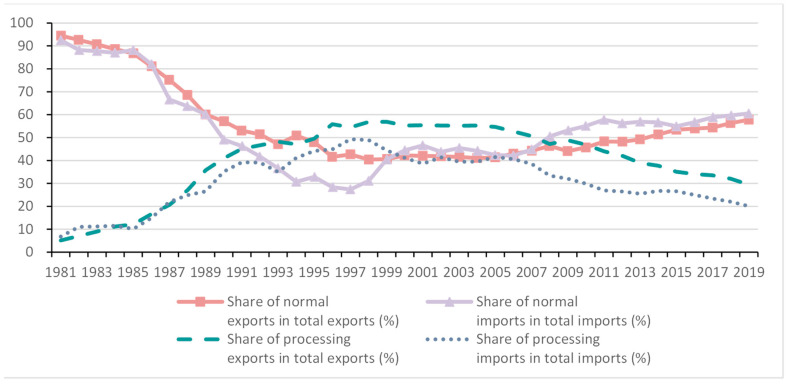
Share of normal and processing trade in China. Source: [14].

Processing exports account for a higher share than normal exports in total merchandise exports during 1995–2010 in China (around 50%, see Figure 1). This share declined especially after the financial crisis in 2008 but remained at a high level. It accounted for about 30% of China’s bilateral trade with the US in 2016. China has an enormous surplus in bilateral trade in goods with developed markets. However, the US exporters and Chinese processing exporters are located at the global production chain’s head and tail, respectively [30]. In the processing exports of some industries in China, the domestic content is as low as 7% [31]. Thus, studies using the traditional competitive I-O and the non-competitive MRIO model, which does not clarify the processing trade, will overstate the embodied emission in exports from China with a high share of processing trade incorporating an important share of imported intermediate components. 

To address these issues, a non-competitive I-O table distinguishing between processing and normal exports has been compiled and used to measure the embodied carbon emissions in Chinese exports [32,33,34,35,36]. However, no study has yet used the non-competitive input-output table distinguishing between processing and normal trade to assess the embodied industrial pollution or carbon emissions in China’s bilateral trade, especially with its major trading partners.

The contributions of this study are as follows. Firstly, a non-competitive I-O model that distinguishes normal and processing exports has been used to avoid over-estimation of embodied emission in exports. We separate these trade patterns to show the various imported intermediate inputs requirements and the high share of processing trade in China. The new evidence from China will also apply for countries with a high percentage of processing trade, such as Mexico or Vietnam. Secondly, while most research focuses on CO_2_ and China’s responsibility for global carbon emissions, this paper examines the increasingly severe industrial wastewater pollutants, waste gas pollutants (including industrial soot, dust, and SO_2_), solid waste pollutants, chemical oxygen demand (COD), and SO_2_ pollution in China and provides policy recommendations. According to the system of discharge fee on various aquatic, atmospheric, and solid pollutants published by China’s Ministry of Environmental Protection (MEP), a new measure called “pollutant equivalent” was designed, and a specific coefficient was assigned to each pollutant to evaluate their respective damage, industrial soot and dust have higher coefficients in air pollutants than other pollutants [37].

This study employs a non-competitive I-O model to estimate the embodied domestic and imported emission of China’s exports, as well as the embodied emission of imports in China’s bilateral trade (with the US, Japan, and Germany. In 2020, China’s exports to these three countries accounted for 26% of total exports, and imports from these three countries accounted for 20% of total imports, according to [38] between 2002 and 2016. We also compute and decompose the balance of embodied emission in trade (*BEET*) and pollution terms of trade (*PTT*). This study reassesses the pollution havens hypothesis (PHH) by examining the embodied emission in China’s bilateral industrial trade with leading trade partners by separating processing and normal exports. Second, it focuses on local industrial pollutants that impact local inhabitants and ecosystems. Third, it presents the *BEET* and *PTT* revisions based on estimating a country’s embodied domestic emission of exports. The effect of processing export and intermediate imports are eliminated. A further decomposition of *BEET* and *PTT* helps to analyze the influences. 

This study answers the following questions. What is the status of industrial pollution transfer in countries with a high proportion of processing trade? Furthermore, what are the driving factors behind the industrial pollution transfer in trade? The remainder of the paper is as follows: Section 2 reviews the existing literature, while Section 3 introduces the methodology and describes the data. Section 4 presents the empirical analysis and discusses the main results. Section 5 concludes the paper. 

## 2. Literature Review

Other things being equal, the PHH states that the degree of environmental regulations will provide a comparative advantage, encouraging polluting industries to relocate from developed countries with stricter environmental regulations to developing countries with more flexible environmental regulations [39,40]. In the empirical test of the PHH, there are two research streams. One of those streams used econometric regression to investigate the existence of the PHH and the influence of environmental policy on investment, industrial production, and trade. The results were inconsistent when different environmental policy indicators, pollutants, periods, and countries were chosen. When the PHH was investigated using econometric regression, the endogeneity of explanatory variables was also significant. The other research stream addresses the concerns raised above by utilizing an I-O model to calculate trade embodied emissions [41]. This does not necessitate a long-term econometric model of time-series data, and the interconnection between different sectors in a country (or between countries) can be illustrated using either a single-region I-O table (SRIO) or a multi-region I-O table (MRIO). The PHH was assessed using the embodied emission in exports minus the embodied emission averted by imports [42,43,44,45,46]. According to [44], there was a rough balance between CO_2_ emissions from China’s exports and emissions saved by its imports between 1997 and 2002. However, ref. [46] found that during 2005 and 2007, emissions in China’s exports were higher than those avoided by imports. 

Based on [47]’s work, further studies [28,48] employ the MRIO to assess embodied emission in final consumption, intermediate trade for the last production stage, and GVC-related trade. Ref. [49] reexamined the PHH by employing the MRIO to quantify the emission intensity of 40 major economies using value-added trade data, demonstrating that high-income countries offshore their emissions to low-income countries by outsourcing pollution-embedded production stages rather than the entire production processes. However, the analyses above do not consider China’s significant processing trade share.

China’s processing exports accounted for 55% of overall exports (trade in goods) in 2002 and declined to 29% in 2019 [14]. The percentage of imported intermediate inputs used in processing export production is substantially higher than normal exports. As a result, processing exports can dramatically increase the imported content for exports compared to domestic use [50]. The non-competitive I-O table, which distinguishes import and domestic intermediate but not processing and normal exports, will overestimate a country’s embodied emissions in exports. In order to solve the issues mentioned above, the non-competitive I-O table distinguishing between processing and normal exports has been compiled [32,33,34,35,36]. Ref. [32] found that the embodied emissions in China’s exports would be overestimated by more than 60% if the processing exports were not separated from the normal exports. Furthermore, processing exports emit 34% less CO_2_ per dollar value than normal exports. Ref. [33] also revealed that the traditional non-competitive I-O model overestimated embodied emission in processing exports while underestimating embodied emission in normal exports, and the embodied CO_2_ emissions in China’s exports would decline by 32% if the non-competitive I-O model differentiating processing and normal exports was used. The study also mentioned the importance of examining the export in embodied emission research for countries with high processing exports like China. Ref. [34] found that embodied CO_2_ emissions were significantly lower in a regionally disaggregated I-O model (distinguishing production and emission efficiency in different regions of China) and an I-O model with a disaggregated export processing sector (separating processing export and normal export) than in a standard model. Ref. [35] revisited the net global CO_2_ transfer using a new “WIOD-DPN” model. They discovered that if China’s processing trade were not classified separately, the net CO_2_ export from China to other regions and from other areas to China would be distorted. At the regional level, ref. [36] established an inter-regional input-output (IRIOP) model that distinguished China’s processing trade from normal trade. They found that the traditional MRIO model overstated China’s environmental impact of exports by 14–25% in 2002 and 7–20% in 2012 for different regions. However, few studies have been conducted to study the embodied pollution transfer in China’s trade with its major trading partners using a non-competitive I-O model that distinguishes between processing trade and normal trade.

Most studies have focused on the pollutant CO_2_ and examined the embodied emission in China’s exports by separating trade patterns [31,32,33,34,35,36], firm ownership [51] and domestic regions [52,53,54,55]. A few studies have focused on PM_2.5_ [18,56] and SO_2_ [53,57]. There is a lack of research on China’s local industrial pollutants, such as COD, soot, dust, and solid waste. 

## 3. Methodology and Data Description

### 3.1. Embodied Emission in Exports with Disaggregated Processing Exports

Ref. [50] first proposed splitting the national I-O table with disaggregated processing exports and investigated the domestic and foreign value-added share of China’s exports. This method was adapted to the extended environmental I-O model by [32,33,34]. This paper constructs a non-competitive input-output model that differentiates between processing exports and normal exports, as well as between domestically produced intermediate inputs and imported intermediate inputs, using detailed data from Chinese customs and the official 42 sectoral competitive input-output tables of China. Notations are listed at the end of the paper. The input was split into domestic and import input, and the final use was divided into domestic use $C^{D}+I^{D}$ (final domestic consumption and capital formation), normal exports $E^{N}$, and processing exports $E^{p}$. The I-O model can be expressed as Equation (1), where the coefficient matrices $A^{DD}$, $A^{DN}$ and $A^{DP}$ represent the domestic intermediate input used for domestic use, normal exports, and processing exports, respectively. $A^{MD}$, $A^{MN}$ and $A^{MP}$ are the coefficient matrices representing the imported intermediate input used for domestic use, normal exports, and processing exports, respectively. $A_{v}^{D}$, $A_{v}^{N}$, and $A_{v}^{P}$ are the value-added coefficient matrices for domestic use, normal exports, and processing exports, respectively. *X* is the total output, *M* is the total imports, and $C^{M}$, $I^{M}$, and $E^{M}$ are the imported products used for final consumption, capital formation, and re-exports, respectively.

(1) $$\begin{array}{l} \left[\begin{array}{ccc} 1-A^{DD} & -A^{DN} & -A^{DP}\\ 0 & 1 & 0\\ 0 & 0 & 1 \end{array}\right]\left[\begin{array}{c} X-E^{N}-E^{P}\\ E^{N}\\ E^{P} \end{array}\right]=\left[\begin{array}{c} C^{D}+I^{D}\\ E^{N}\\ E^{P} \end{array}\right]\\ A^{MD}\left(X-E^{N}-E^{P}\right)+A^{MN}E^{N}+A^{MP}E^{P}+C^{M}+I^{M}+E^{M}=M\\ uA^{DD}+uA^{MD}+A_{v}^{D}=u\\ uA^{DN}+uA^{MN}+A_{v}^{N}=u\\ uA^{DP}+uA^{MP}+A_{v}^{P}=u \end{array}$$

The total input coefficient matrix can be obtained as follows:

(2) $$B={\left[\begin{array}{ccc} 1-A^{DD} & -A^{DN} & -A^{DP}\\ 0 & 1 & 0\\ 0 & 0 & 1 \end{array}\right]}^{-1}=\left[\begin{array}{ccc} {\left(1-A^{DD}\right)}^{-1} & {\left(1-A^{DD}\right)}^{-1}A^{DN} & {\left(1-A^{DD}\right)}^{-1}A^{DP}\\ 0 & 1 & 0\\ 0 & 0 & 1 \end{array}\right]$$

Substituting Equation (2) into Equation (1), we obtain:

(3) $$X-E^{N}-E^{P}={\left(1-A^{DD}\right)}^{-1}\left(C^{D}+I^{D}\right)+{\left(1-A^{DD}\right)}^{-1}A^{DN}E^{N}+{\left(1-A^{DD}\right)}^{-1}A^{DP}E^{P}$$

Further substituting Equation (3) into the second equation of Equation (1), we get the total demand equation for the imported intermediate goods as follows:

(4) $$\begin{array}{l} M-C^{M}-I^{M}-E^{M}=A^{MD}{\left(1-A^{DD}\right)}^{-1}(C^{D}+I^{D})+A^{MD}{\left(1-A^{DD}\right)}^{-1}A^{DN}E^{N}\\ +A^{MD}{\left(1-A^{DD}\right)}^{-1}A^{DP}E^{P}+A^{MN}E^{N}+A^{MP}E^{P} \end{array}$$

The imported intermediate goods can be divided into use for domestic consumption and investment, indirect normal export production, indirect processing export production, direct normal export production, and direct processing export production.

The total import value of a country’s exports per unit can be expressed as the sum of the total import value in normal and processing exports, as shown in Equation (5):

(5) $$TVSS=\left(A^{MD}{\left(1-A^{DD}\right)}^{-1}A^{DN}+A^{MN}\right)\frac{E^{N}}{E}+\left(A^{MD}{\left(1-A^{DD}\right)}^{-1}A^{DP}+A^{MP}\right)\frac{E^{P}}{E}$$

The total domestic value-added coefficient of unit domestic consumption, normal exports, and processing exports can be expressed as follows:

(6) $${\left[\begin{array}{l} \begin{array}{c} DVS^{D}\\ DVS^{N} \end{array}\\ DVS^{P} \end{array}\right]}^{T}={\bar{A}}_{v}B=\left[\begin{array}{ccc} A_{v}^{D} & A_{v}^{N} & A_{v}^{P} \end{array}\right]\times \left[\begin{array}{ccc} {\left(1-A^{DD}\right)}^{-1} & {\left(1-A^{DD}\right)}^{-1}A^{DN} & {\left(1-A^{DD}\right)}^{-1}A^{DP}\\ 0 & 1 & 0\\ 0 & 0 & 1 \end{array}\right]={\left[\begin{array}{c} A_{v}^{D}{\left(1-A^{DD}\right)}^{-1}\\ A_{v}^{D}{\left(1-A^{DD}\right)}^{-1}A^{DN}+A_{v}^{N}\\ A_{v}^{D}{\left(1-A^{DD}\right)}^{-1}A^{DP}+A_{v}^{P} \end{array}\right]}^{T}$$

Further, the total domestic value of a country’s exports per unit can be expressed as below:

(7) $$TDVS=\left(A_{v}^{D}{\left(1-A^{DD}\right)}^{-1}A^{DN}+A_{v}^{N}\right)\frac{E^{N}}{E}+\left(A_{v}^{D}{\left(1-A^{DD}\right)}^{-1}A^{DP}+A_{v}^{P}\right)\frac{E^{P}}{E}$$

The embodied domestic emissions in normal and processing exports are presented in Equation (8), and the embodied imported emissions from normal and processing exports are shown in Equation (9). Equations (8) and (9) are obtained by multiplying Equations (7) and (5) by the direct emission intensity *EMI/X*, respectively, μ is the unit vector.

(8) $$EEX_{domestic}=\left(A_{v}^{D}{\left(1-A^{DD}\right)}^{-1}A^{DN}+A_{v}^{N}\right)\frac{E^{N}}{X}EMI+\left(A_{v}^{D}{\left(1-A^{DD}\right)}^{-1}A^{DP}+A_{v}^{P}\right)\frac{E^{P}}{X}EMI$$

(9) $$EEX_{imported}=\mu \left(A^{MD}{\left(1-A^{DD}\right)}^{-1}A^{DN}+A^{MN}\right)\frac{E^{N}}{X}EMI+\mu \left(A^{MD}{\left(1-A^{DD}\right)}^{-1}A^{DP}+A^{MP}\right)\frac{E^{P}}{X}EMI$$

### 3.2. The Balance of Embodied Emission in Trade (BEET)

*BEET* is defined as embodied emission in imports (*EIM*) minus embodied emission in exports (*EEX*). However, because the imported intermediate input has no impact on the environment of the importing country, the emissions embodied in the imported intermediate input should be excluded from the *BEET* calculation. We replace *EEX* with embodied domestic emission in export (*EEX_domestic_*) for China and define the revised *BEET* in Equation (10). Suppose the pollution avoided from imports is less than the pollution produced by exports. In that case, the country is an environment deficit country (net pollution exporter) and bears a heavier burden of pollution-intensive product manufacturing in the global division. If *BEET* > 0, the country is an environment surplus country (net pollution importer), meaning it avoids more pollution from imports than it produces for exports; other countries bear the brunt of pollution-intensive product manufacturing.

(10) $$BEET=EIM-EEX_{domestic}=c^{*}L^{*}M-c\left\{A_{v}^{D}{\left(I-A^{DD}\right)}^{-1}A^{DN}+A_{V}^{N}\right\}E^{N}-c\left\{A_{v}^{D}{\left(I-A^{DD}\right)}^{-1}A^{DP}+A_{V}^{P}\right\}E^{P},$$

where *c* and c* (due to limitation of data, we adopted an alternative method by [58] to estimate the direct emission intensity of corresponding pollutants in the US, Japan, and Germany, $c_{ij}^{k}=h_{ij}^{k}*\left(L_{ij}/X_{ij}\right)$, where $c_{ij}^{k}$ is the direct emission intensity where the denominator is the output, $h_{ij}^{k}$ is the direct emission intensity where the denominator is the labor force, $L_{ij}/X_{ij}$ is the ratio of the labor force to output, k represents different pollutants, i is the sector, and j is the country. According to [58], the differences of $h_{ij}^{k}$ between countries are generally small (developing countries produce more pollutants but have more workers). Therefore, this paper estimates the direct emission intensity of corresponding pollutants in the US, Japan, and Germany through the above equation indirectly, and $h_{ij}^{k}$ is calculated based on Chinese historical data) are the direct emission intensities for China and foreign countries, respectively, and L* is the foreign country’s Leontief inverse matrix. 

### 3.3. The Pollution Terms of Trade (PTT) 

The conventional *PTT* index is calculated by dividing the embodied emission in exports per unit value by the embodied emission in imports per unit value. We modify *PTT* per Equation (11) to separate the processing exports and eliminate the effect of imported intermediate input. If *PTT* > 1, exports are more polluting than imports, and other countries have transferred environmental costs to the exporting country. If *PTT* < 1, a country’s imports are more polluting than its exports, and the environmental costs are transferred to foreign countries.

(11) $$PTT=\frac{EEX_{domestic}}{X}/\frac{EIM}{M}=\frac{c\left\{A_{v}^{D}{\left(I-A^{DD}\right)}^{-1}A^{DN}+A_{V}^{N}\right\}E^{N}+c\left\{A_{v}^{D}{\left(I-A^{DD}\right)}^{-1}A^{DP}+A_{V}^{P}\right\}E^{P}}{E^{N}+E^{P}}/\frac{c^{*}L^{*}M}{M}$$

### 3.4. Decomposition of the BEET and PTT

This study decomposes the change in *BEET* from 2002 to 2016 into three factors, including the scale effect, structural effect, and technical effect, similar to [59]. $\;\Delta BEET/BEET^{2002}=\left(BEET^{2016}-BEET^{2002}\right)/BEET^{2002}*100$, where the three effects are measured by various values of $BEET^{2016}$. To calculate the scale effect, we use $BEET^{2016}$, which assumes that the trade structure (import share, normal and processing export share of each sector), as well as technology (total emission intensity), remain constant (as of 2002), and that only the export scale varies (taking the value in 2016). To compute the structural effect, we use $BEET^{2016}$, which assumes that the export scale and technology remain constant (based on the value in 2002) and only the trade structure changes (based on the value in 2016). Finally, to measure the technical effect, we calculate $BEET^{2016}$ assuming that the export scale and trade structure remain unchanged (using the value in 2002), and only the technique (total emission intensity) changes (taking the value in 2016).

Similarly, the structural and technical effects of the *PTT* value change from 2002 to 2016 are decomposed.$\;\Delta PTT/PTT^{2002}=\left(PTT^{2016}-PTT^{2002}\right)/PTT^{2002}*100$, and the two effects were assessed for various values of $PTT^{2016}$. To compute the structural effect, we calculated $PTT^{2016}$ assuming no changes in technology (using the value from 2002) and just changes in the export structure (taking the value in 2016). To compute the technical effect, we calculated $PTT^{2016}$, assuming that the trade structure remains constant (using the value from 2002) and only the technique (total emission intensity) changes (taking the value in 2016).

*BEET* is determined by the trade balance, emission intensity, and export/import structure, and the sign can be easily influenced by the trade balance [60], whereas *PTT* is unaffected by the trade balance and can reflect the relative pollution of exports versus imports in a country. The *BEET* analyzes the reverse flow of emissions embodied in a country’s imports and exports, as well as the whole impact of trade on the environment, including scale, structural, and technical effects. *PTT* focuses primarily on structural and technical effects.

### 3.5. Data Description

The Chinese official I-O tables for 42 sectors were obtained from the National Bureau of Statistics of China (due to the compilation system, the input-output table of China is compiled for the years ending in 2 and 7, and the extended table (projection table) is compiled for the years ending in 0 and 5. Therefore, this paper estimates the input-output coefficients of 2016 with the official extension table published in 2015). The I-O tables for the US, Japan, and Germany were from the Organization for Economic Co-operation and Development (OECD) database. We obtained detailed bilateral trade data from China’s General Administration of Customs and the exchange rates from International Financial Statistics. The output and labor force of different sectors in each country were obtained from the OECD Structural Analysis (STAN) database. Industrial emissions data were from the *China Environmental Yearbook*. As shown in Appendix A, these sectors were combined into 15 manufacturing sectors. The pollutants examined in this study include industrial wastewater, waste gas, solid waste pollutants, chemical oxygen demand (COD), and sulfur dioxide (SO_2_) in tons. Wastewater, waste gas, and solid waste pollutants are the sum of the pure weight of pollutant emissions from production.

## 4. Empirical Results and Analysis

### 4.1. The Embodied Emission in China’s Bilateral Trade 

#### 4.1.1. The Embodied Emission in Exports

There are two types of embodied emissions in exports: Domestic and imported.

**Figure 2:**
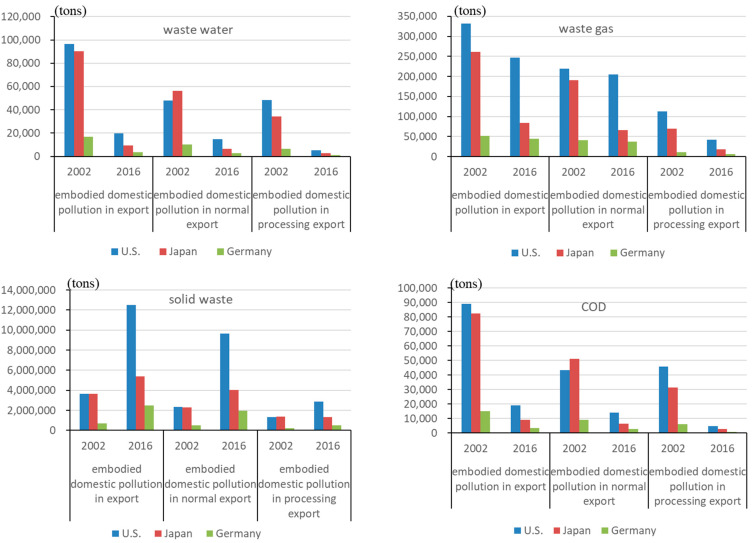

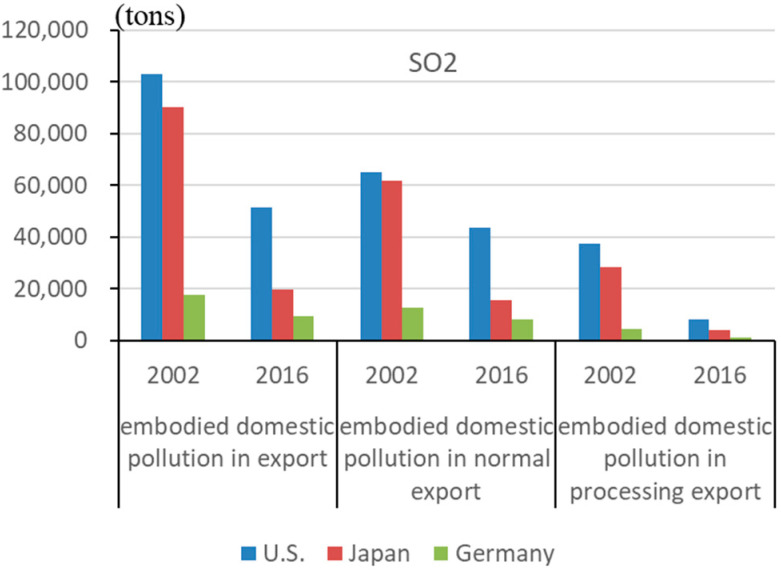
The embodied domestic emission in China’s exports to the US, Japan, and Germany (unit: tons).

*The embodied domestic emission in exports.*Figure 2 depicts the embodied domestic emission in China’s exports to the US, Japan, and Germany. Because of the scale of bilateral trade, domestic pollutant emissions from China’s exports to the US are the highest, surpassing exports to Japan and Germany. Domestic wastewater pollutants (including COD) caused by exports to the US, Japan, and Germany declined dramatically. China has achieved great success in the abatement of wastewater in each sector, with the rise of industry concentration and significant improvements in pollution control capacity. From 2002 to 2016, the wastewater emission intensity of all manufacturing industries decreased by an average of 93%, higher than that of waste gas (73%) and solid waste (18%). The number of industrial wastewater treatment facilities and treatment capacity in China increased by 135% and 27%, respectively, from the first National Survey of Pollution Sources (2007) to the second National Survey of Pollution Sources (2017). Desulfurization and dust removal facilities grew by 227% and 402%, respectively. However, solid waste pollutants increased significantly, in which normal exports contributed to 83%, 100% and 84%, respectively. From 2002 to 2016, the share of normal exports in total manufacturing exports (normal and processing exports) to the US, Japan, and Germany increased from 32%, 38%, and 38% to 58%, 51%, and 60%, respectively, thanks to learning-by-processing exporting [61], whereas the share of processing exports decreased. Due to the high solid waste emission intensity (iron and steel, non-ferrous metals ranked first, and the chemical industry ranked second) and normal exports share (around 10%), the chemical industry generated the highest domestic solid waste emissions (about 40%) among export industries and accounted for the highest proportion of the total increase in domestic solid waste emission among China’s exports to the US, Japan, and Germany in 2016 (34%, 73%, and 40% respectively). The domestic solid waste from exports increased at a much faster rate than the average in the wood processing and furniture manufacturing industry, metal products industry, communication equipment, and computer and other electronic equipment manufacturing industry, owing to the rapid increase in the normal exports share of these three industries. The above three sectors accounted for 34%, 45%, and 27% of the domestic manufacturing solid waste emissions caused by exports to the US, Japan, and Germany.

Normal exports are still the main trade pattern causing domestic pollution; however, the extent to which different trade patterns play a role in the decline of wastewater and waste gas pollutants is not consistent. The decrease in domestic wastewater and waste gas emissions from exports to the US was mostly attributable to a decrease in processing exports, which accounted for 57% and 83%, respectively. Normal exports accounted for 61% and 71% of the decline in domestic wastewater and waste gas emissions from exports to Japan. Normal exports accounted for 57% of the decline in domestic wastewater emissions generated by exports to Germany, and processing exports accounted for 64% of the decline in domestic waste gas emissions.

**Figure 3:**
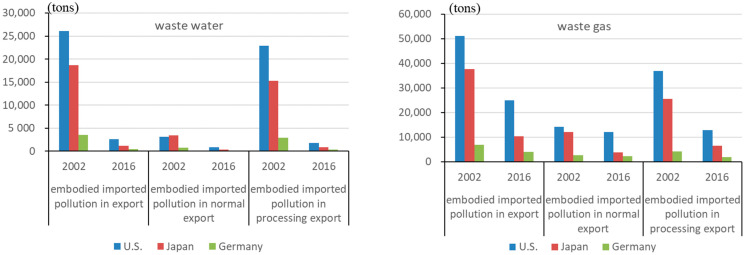

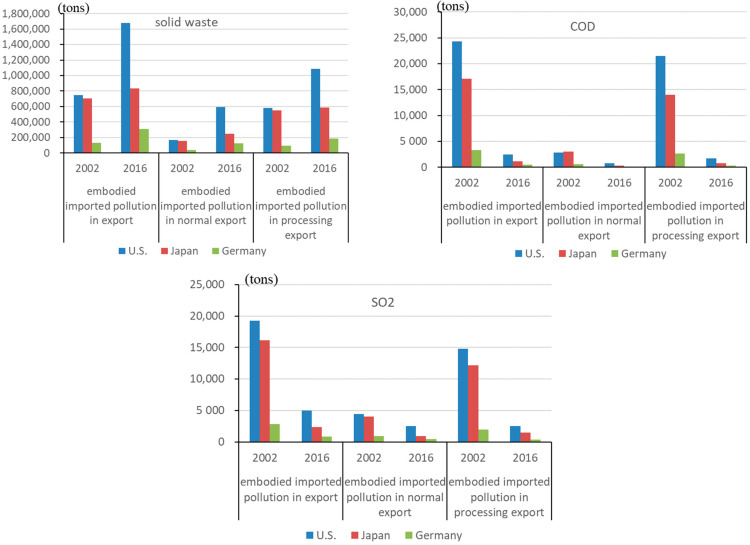
The embodied imported emission in China’s exports to the US, Japan, and Germany (unit: tons).

*The embodied imported emission in exports.*Figure 3 depicts the embodied imported pollution in China’s exports to the US, Japan, and Germany. Similarly, China ranked first in terms of imported emissions in exports to the US, followed by Japan and Germany. Processing exports require more imported intermediate inputs than normal exports; hence they have a significant impact on the change in imported emissions in exports. While avoided wastewater (including COD) and waste gas (including SO_2_) emissions have fallen in China’s exports, avoided solid waste emissions have risen. About 70% of solid waste pollution was avoided by the chemical industry, metal smelting and rolling industries (both have high solid waste emission intensity), and the manufacturing of communications equipment, computers, and other electronic equipment (high processing exports share).

#### 4.1.2. The Embodied Emission in Imports

**Figure 4:**
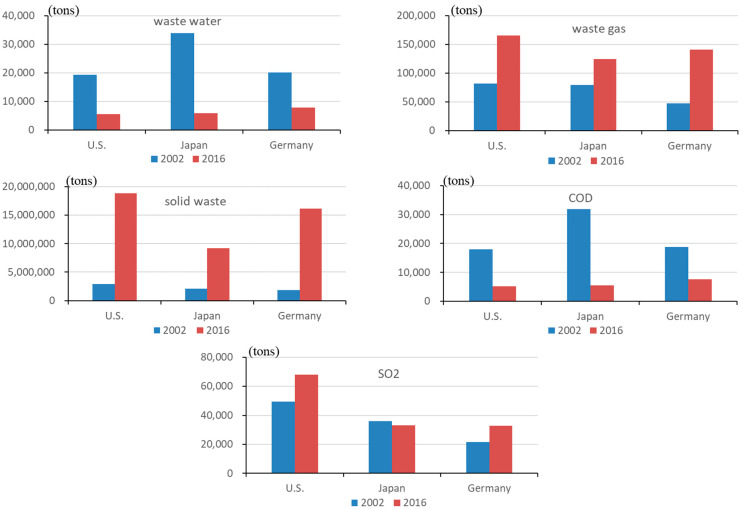
The embodied emission in China’s imports from the US, Japan, and Germany (unit: tons).

The embodied emission in China’s imports from the US, Japan, and Germany is depicted in Figure 4. In contrast to the decrease in wastewater and COD embodied in imports, the solid waste and waste gas (including SO_2_) embodied in China’s imports have increased dramatically from these countries. The main reason is an increase in solid waste emission intensity, as well as a considerable increase in the import proportion of several industries (such as wood and products of wood and cork, manufacture of furniture, coke, refined petroleum products, nuclear fuel, other non-metallic mineral products, and transportation equipment). Overall, sectors with high import shares from the US, Japan, and Germany have low emission intensities; however, sectors with high emission intensities have low import shares, such as China’s import proportions of iron and steel and non-ferrous metals, with the highest solid waste emission intensity from the US, Japan, and Germany, were only 4%, 8%, and 2%, respectively, in 2016. China’s domestic pollution can be reduced in the future by changing its import mix.

### 4.2. The Revised BEET 

#### 4.2.1. Revised *BEET*

**Figure 5:**
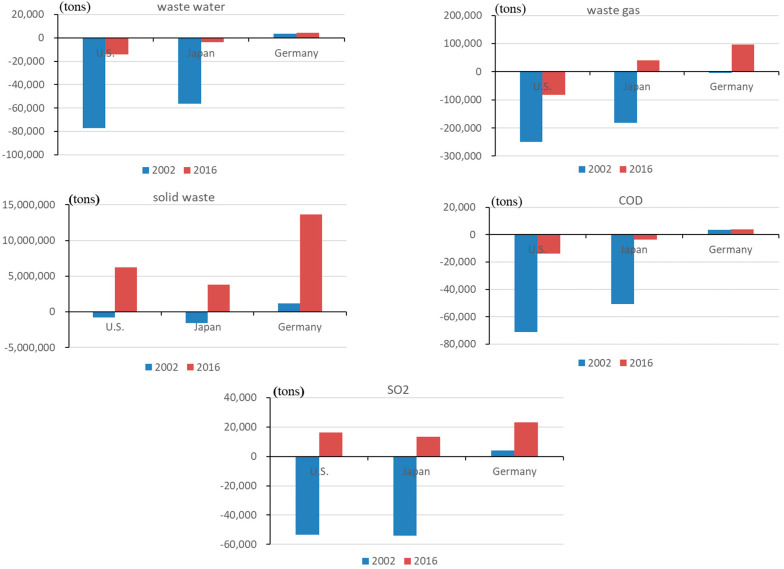
The revised *BEET* between China and US, Japan, and Germany (unit: tons).

Figure 5 depicts the revised balance of embodied emissions in China’s bilateral trade. As a result of the increasingly strict environmental protection in China and the adjustment of its trade structure, the environmental cost borne by China from production for export to developed countries has decreased to varying degrees, which is reflected in the gradual reduction of the pollution deficit (the embodied emission in imports is less than the domestic emission caused by exports) or increase in pollution surplus (the domestic emission caused by exports is less than the embodied emission in imports). China bore more pollution in its bilateral trade with the US and Japan in 2002 than in 2016. However, it became an SO_2_ and solid waste pollution surplus country in its trade with the US and a waste gas (including SO_2_) and solid waste pollution surplus country in its trade with Japan. In its trade with Germany, China had a pollution surplus, and the surplus was growing.

The decrease in pollution deficit (or increase in pollution surplus) is substantially greater in solid waste and SO_2_. Between 2002 and 2016, China’s pollution deficits in solid waste, SO_2_, wastewater, COD, and waste gas in bilateral trade with the US decreased by 918%, 131%, 81%, 81%, and 67%, respectively. They have been decreased by 340%, 125%, 94%, 93%, and 122% in bilateral trade with Japan, while the pollution surplus in bilateral trade with Germany increased by 1085%, 463%, 25%, 10%, and 2218%. Bilateral trade between China and the US has the most detrimental influence on China’s local environment, followed by bilateral trade between China and Japan.

#### 4.2.2. Sector Analysis of the Revised *BEET*

In 2016, the proportion of revised *BEET* by sector was shown in Table 1 ($BEET_{i}/\underset{i}{\sum }BEET_{i}$, where *i* indicates sector). Depending on the sign of the overall *BEET* value, the sign of each sector (“+” or “–”) has distinct implications. If the total *BEET* value is positive, sectors with positive values have a pollution surplus; they avoid more pollution from imports than they produce for exports. In contrast, sectors with negative values have a pollution deficit; they produce more pollution for exports than they avoid by imports. If the total *BEET* value is negative, sectors with negative values have a pollution surplus, while sectors with positive values have a pollution deficit.

In the bilateral trade between China and the US, sectors 2, 3, 4, 5, 7, and 8 have pollution deficits in all pollutants. Sectors 2, 3, 5, and 7 were the sectors with large wastewater (including COD) deficits. Sectors 8, 7, 4, and 2 were the sectors with major waste gas (including SO_2_) deficits, while 7, 4, and 8 were the sectors with considerable solid waste deficits. While pollution deficits (surpluses) in most sectors declined (raised), wastewater (COD) pollution deficits in sectors 14 and 15, waste gas (including SO_2_) pollution deficits in sectors 3, 4, and 10, and solid waste pollution deficits in sectors 10 and 4 increased. 

In the bilateral trade between China and Japan, sectors 1, 2, 3, 4, 5, and 6 had pollution deficits in all pollutants. Sectors 1, 2, and 3 were the sectors with major wastewater (including COD) deficits, while 8, 4, 1, and 2 were the sectors with considerable waste gas (including SO_2_) deficits. Sectors 7 and 1 were the sectors with major solid waste deficits. In most sectors, the pollution deficit (surplus) was reducing (growing). However, the wastewater (including COD) surplus decreased in sectors 9, 10, 11, 13, 14, and 15, the waste gas pollution deficit increased in sector 4, and the solid waste pollution deficit increased in sector 5.

In the bilateral trade between China and Germany, sectors 2, 3, and 4 had pollution deficits in all pollutants, sectors 5 and 8 had waste gas (including SO_2_) deficits, and sectors 5 and 14 had wastewater (including COD) deficits. Sector 2 had a significant wastewater (including COD) deficit, whereas sector 8 had a considerable waste gas (including SO_2_) deficit. The pollution deficit (surplus) in most sectors was reducing (growing). While the waste gas and solid waste deficits grew in sector 3, the surplus of wastewater (including COD) reduced in sectors 4, 5, 9, 10, 11, 12, 13, 14, and 15, and the surplus of waste gas (including SO_2_) and solid waste declined in sectors 4 and 5.

Overall, the deficit industries are concentrated in sectors 2, 3, 4, 5, and 8. This has to do with the bilateral trade structure. The industries above accounted for 44%, 42%, and 38% of China’s normal exports to the US, Japan, and Germany, respectively, and 16%, 15%, and 8% of China’s processing exports, respectively, while only 13%, 6%, and 3% of China’s imports came from the US, Japan, and Germany. Meanwhile, manufacturing industries with low emission intensities (11, 12, 13, 14, 15) are primarily exported in the form of processing exports, which has a lower impact on domestic pollution than normal exports (accounting for 78%, 74%, and 85% of processing exports to the US, Japan, and Germany, respectively, among which the processing exports of industry 14 accounted for about 40%). China can continue to optimize its import and export structures in the future by increasing the share of low (high) pollution intensity sectors’ exports (imports) and decreasing the share of high pollution intensity industries’ exports to minimize the negative environmental impact of trade.

#### 4.2.3. The Effect Decomposition of Revised *BEET*

From 2002 to 2016, Table 2 demonstrates the effect decomposition of the revised *BEET* on China’s bilateral trade with the US, Japan, and Germany. The scale effect exacerbated China’s pollution deficits in bilateral trade with the US and Japan. However, both the reduction in overall emission intensity (technological improvement effect) and the adjustment in trade structure (structural shift) substantially decreased the negative impact of trade on China’s local environment, where technical effects play a key role. However, the scale, structural, and technical effects vary with different pollutants in the bilateral trade between China and Germany.

### 4.3. Revised PTT 

#### 4.3.1. Revised *PTT* in Bilateral Trade 

**Figure 6:**
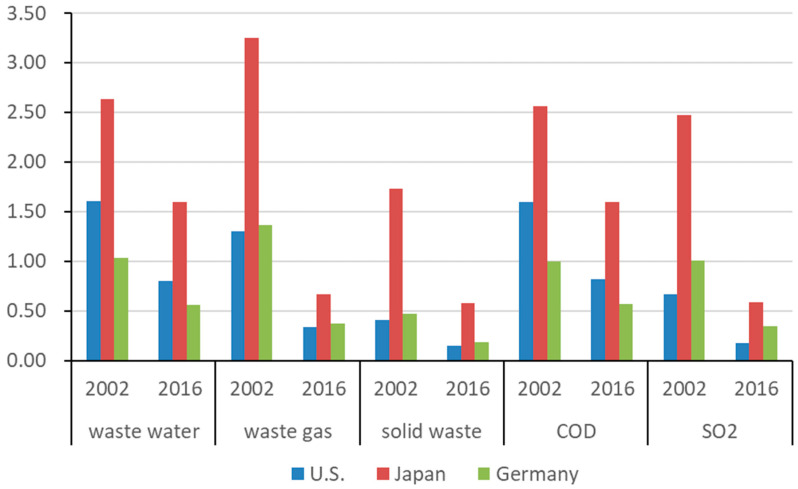
The revised *PTT* between China and US, Japan, and Germany.

Figure 6 depicts China’s revised *PTT* in bilateral trade with the US, Japan, and Germany. Compared to *BEET*, *PTT* can indicate the relative pollution of a country’s exports and is not affected by the trade balance. Unlike *BEET*’s calculation that China bears the greatest environmental pollution costs for its exports to the United States, China’s exports per unit to Japan are the most polluting, followed by the United States in terms of wastewater (COD) and Germany in terms of waste gas (SO_2_) and solid waste pollution. Except for solid waste and SO_2_ (*PTT*_solid waste_ = 0.41, *PTT*_SO2_ = 0.67), China’s exports to the US were more polluting than its imports from the US in 2002. After the influence of imported intermediate input was removed, the exports were less polluting than imports in 2016. China’s exports to Japan were more polluting than its imports from Japan in 2002, especially for waste gas and wastewater (*PTT*_waste gas_ = 3.25, *PTT*_wastewater_ = 2.63). In 2016, its exports to Japan were less polluting than imports in the waste gas, SO_2_, and solid waste (*PTT*_waste gas_ = 0.67, *PTT*_SO2_ = 0.59, *PTT*_solid waste_ = 0.58). However, in terms of wastewater and COD, its exports were still more polluting than imports (the *PTT* values were 1.59 and 1.60, respectively). Except for solid waste (*PTT*_solid waste_ = 0.47), China’s exports to Germany were slightly more polluting than its imports from Germany in 2002 but were less polluting than its imports from Germany in 2016. The relative pollution of exports fell the most in exports to the US, followed by Japan and Germany. In terms of pollutants, the relative pollution of exports in the waste gas, SO_2_, and solid waste fell the most, followed by wastewater and COD pollutants. 

#### 4.3.2. The Effect Decomposition of Revised *PTT*

The effect decomposition of the revised *PTT* in China’s bilateral trade with the US, Japan, and Germany is shown in Table 3. The structural effect, and especially the technical effect, lowered the relative pollution of China’s exports to the US, Japan, and Germany, particularly in waste gas (including SO_2_) and solid waste.

## 5. Conclusions

The PHH was tested in China-U.S. and China-Japan bilateral trade in 2002 but not in 2016, when processing exports were taken into account. China’s bilateral trade with the US had the most detrimental influence on China’s local environment, followed by China’s bilateral trade with Japan. In 2002, China had a pollution deficit in bilateral trade with the US and Japan [26,28,29], and in 2016, China had a pollution surplus with the US for solid waste and SO_2_, as well as a pollution surplus with Japan for waste gas, solid waste, and SO_2_. Furthermore, in most pollutants, China had a pollution surplus with Germany between 2002 and 2016. According to the calculation of *PTT*, exports to Japan are more polluting per unit than those to the US and Germany.

As a result of China’s exceptional accomplishment in reducing wastewater pollution, the amount of domestic wastewater pollutants (including COD) caused by exports has greatly decreased. However, solid waste pollution is increasing, partially because the intensity of solid waste emissions in each sector has not fallen as much as wastewater (including COD) and waste gas (including SO_2_), and partly because China’s normal exports to the US and Germany have increased.

China has avoided domestic emissions by using imported intermediate inputs primarily through processing imports. The avoided wastewater (including COD) and waste gas (including SO_2_) embodied in exports to the US, Japan, and Germany have declined, mainly caused by the decline in processing exports, while solid waste is rising, which is mostly due to the rise in the share of normal exports and the relatively high solid waste emission intensity. The effect decomposition of the revised *BEET* and *PTT* demonstrate that the negative environmental impact of bilateral trade with the US and Japan is declining in China. This can be attributed to technical and structural transformations toward more high-tech, less polluting, as well as more value-added green industrial productions [26,29,62,63]. The sector analysis of the revised *BEET* demonstrates that the negative impact of trade on China’s local environment is gradually diminishing. However, several sectors are still in a pollution deficit due to the high proportion of normal exports. 

The following policy recommendations are made in this paper to improve the sustainable development of trade between China, the US, Japan, and Germany. It makes recommendations for reducing the negative environmental impact of bilateral trade between developed and developing countries under the GVC.

China should continue to adjust its trade structure (especially normal exports) and expand imports, increasing the import share of iron and steel, non-ferrous metals, and chemical industries with high solid waste emission intensity. Efforts should be made to reduce the export share (mainly normal exports) and increase the import share of sectors with significant pollution deficits, such as textiles, textile products, leather, down, and footwear, wood and products of wood and cork, furniture manufacturing, pulp, paper, paper products, printing and publishing, stationery manufacturing, and other non-metallic mineral products.

1. The domestic environmental impact of increased normal exports should be prioritized. With the improvement of firms’ learning ability through processing trade after the reform and opening up [61], industries with a high proportion of processing trade, such as textiles, have gradually shifted from processing exports to normal exports while the normal and processing export shares of relatively clean industries, such as machinery and equipment manufacturing, have increased. Normal exports consume more domestic intermediate inputs than processing exports, resulting in a greater impact on domestic pollution. As a result, environmental regulations should be tightened even further to mitigate the negative consequences of increased normal exports on the domestic environment.
2. China should continue to reduce emissions intensity, particularly solid waste emissions, and close the gap with developed countries [64]. China should boost productivity by stimulating innovation and expanding R&D spending, as well as seek to reduce pollution by raising environmental standards and implementing strong environmental laws [37].

The study’s policy implications are that global industrial fragmentation is altering global trade patterns and making measuring the environmental cost generated by international trade more difficult. Developing country governments should strike a balance between economic development and environmental conservation as they integrate into the GVC. Processing trade should be taken into account when assessing the environmental cost of trade for nations with a large percentage of processing trade. As China transitions from processing to normal exports and processing exports become more concentrated in relatively clean industries, environmental challenges resulting from normal exports should be given greater consideration.

The current and next 20 years will be an important period for China’s rapid socio-economic development. Industrialization will remain the main driver of China’s economic development, and coal, chemical, steel, petrochemical, paper, pharmaceutical, leather and metallurgical industries will remain the basic industries in China, supporting the overall development of China’s industrial production and possibly leading the international development in this field. China’s future high economic growth means more serious environmental challenges. In the context of the rise of a new round of industrial revolution, new industries and new models marked by green, cloud computing, and smart manufacturing bits will play a key role in development. The strategy of industrial pollution control should gradually change from end-to-end treatment to source and whole process control of industrial production. Particular attention should be paid to the whole process of control of toxic and harmful pollution in key industries, gradually shifting from a single field of environmental treatment to multi-media collaborative pollution control and regional coordination of pollution control, improving the collaborative monitoring capability of pollutants, enhancing the research and development of pollution control technology and financial support, and strengthening the cooperation between industry, academic and research to promote green industrial development.

Future research could be expanded in numerous ways. First, the direct emission intensity of imported products was estimated to be comparable to China’s industrial pollutants; future studies can examine pollution data more precisely using consistent criteria. Second, this study employs a non-competitive I-O table that distinguishes between processing and normal exports. Future studies are expected to provide a comprehensive understanding of the pollution caused by trade under the GVC, particularly for countries like China and Vietnam, by employing an MRIO model that incorporates processing export.

## Figures and Tables

**Table 1:** The proportion of revised sectoral *BEET* between China and the US, Japan, and Germany (2016, unit for each sector: %; unit for total: tons).

Sector	US					Japan					Germany				
	Wastewater	Waste Gas	Solid Waste	COD	SO_2_	Wastewater	Waste Gas	Solid Waste	COD	SO_2_	Wastewater	Waste Gas	Solid Waste	COD	SO_2_
1	**4.22**	−0.53	1.12	**4.23**	0.62	**66.99**	**−10.85**	**−11.69**	**66.92**	**−13.03**	49.10	5.07	4.11	49.78	7.40
2	**42.43**	**11.82**	**−6.58**	**42.61**	**−25.76**	**59.71**	**−5.82**	**−2.49**	**59.97**	**−10.07**	**−23.69**	**−1.23**	**−0.11**	**−24.15**	**−2.67**
3	**12.51**	**18.30**	**−2.73**	**12.34**	**−10.58**	**16.63**	**−12.63**	**−1.64**	**16.37**	**−4.39**	**−7.09**	**−2.58**	**−0.18**	**−7.09**	**−1.24**
4	**5.02**	**41.80**	**−21.85**	**5.24**	**−31.31**	**4.11**	**−17.49**	**−7.98**	**4.27**	**−8.31**	**−0.80**	**−2.38**	**−0.44**	**−0.88**	**−1.41**
5	**11.76**	**3.79**	**−2.80**	**12.02**	**−7.39**	**6.88**	**−1.03**	**−0.69**	**7.04**	**−1.41**	**−2.83**	**−0.21**	0.01	**−2.93**	**−0.41**
6	−0.15	−1.02	3.10	−0.15	1.00	**0.49**	**−2.39**	**−1.83**	**0.45**	**−1.81**	0.48	0.97	1.37	0.49	0.42
7	**14.76**	**22.77**	**−43.98**	**13.96**	**−37.66**	−2.86	5.33	**−10.71**	−3.52	**−0.32**	21.38	12.24	13.13	21.05	13.01
8	**0.92**	**103.47**	**−16.65**	**0.94**	**−111.64**	−0.75	**−13.75**	**−1.31**	−0.74	**−8.88**	0.42	**−4.88**	1.63	0.40	**−4.56**
9	−5.72	−56.02	86.68	−5.45	139.10	−9.79	44.87	48.31	−9.54	49.03	3.53	11.97	14.32	3.45	15.49
10	**4.49**	**16.34**	**−1.50**	**4.48**	9.12	**0.76**	0.79	4.00	**0.76**	4.42	3.38	6.11	5.37	3.37	5.87
11	−0.58	−6.99	27.75	−0.48	42.49	−12.30	27.29	21.81	−12.19	21.87	17.16	22.92	16.01	17.25	18.78
12	−2.46	−33.83	52.39	−2.28	77.73	−10.41	24.45	20.62	−10.32	19.14	31.06	39.19	33.66	31.23	35.86
13	**3.46**	−3.45	9.58	**3.43**	14.31	−6.69	22.00	19.96	−6.61	21.45	5.92	7.49	6.84	5.91	7.94
14	**8.47**	−8.16	3.25	**8.19**	21.67	−3.87	25.37	12.83	−4.06	21.18	**−2.63**	2.42	1.51	**−2.51**	2.76
15	**0.85**	−8.28	12.23	**0.94**	18.30	−8.90	13.86	10.82	−8.79	11.14	4.60	2.90	2.77	4.63	2.75
**Total**	−14,320.94	−81,799.56	6,261,477.50	−13,764.00	16,336.79	−3589.40	40,557.93	3,769,664.76	−3453.54	13,419.01	4212.15	97,065.22	13,675,997.63	3992.22	23,253.55

Note: See the sector classification in Appendix A. Sectors with pollution deficits are bold.

**Table 2:** The effect decomposition of revised *BEET* (unit: %).

		Wastewater	Waste Gas	Solid Waste	COD	SO_2_
US	Scale effect	432.27	551.10	1458.24	426.77	692.41
	Structural effect	−19.56	−34.02	−72.39	−20.21	−35.05
	Technical effect	−96.57	−99.79	−594.53	−96.41	−123.57
Japan	Scale effect	152.37	166.28	177.03	152.45	166.68
	Structural effect	−15.85	−35.34	−13.14	−16.12	−27.60
	Technical effect	−96.97	−136.98	−4458.98	−97.06	−112.90
Germany	Scale effect	−197.60	2318.69	247.23	−85.35	−326.33
	Structural effect	24.40	−133.86	−4.39	11.81	19.42
	Technical effect	−82.38	−666.84	201.88	−84.59	49.77

**Table 3:** The effect of decomposition of revised *PTT* (unit: %).

		Wastewater	Waste Gas	Solid Waste	COD	SO_2_
US	Structural effect	−20.88	−25.98	−15.04	−21.55	−17.76
	Technical effect	−52.18	−75.08	−65.07	−51.12	−76.40
Japan	Structural effect	−3.89	−22.91	−2.89	−4.08	−13.11
	Technical effect	−44.48	−55.05	−15.71	−42.98	−40.92
Germany	Structural effect	−8.57	−12.03	−8.77	−7.15	−7.82
	Technical effect	−28.97	−76.80	−63.51	−26.44	−70.15

## Data Availability

Not applicable.

## Notations

Notation	Meaning
$C^{D}$	Final domestic consumption
$I^{D}$	Final capital formation
$E^{N}$	Normal exports
$E^{p}$	Processing exports
$A^{DD}$	Coefficient matrix of domestic intermediate input used for domestic use
$A^{DN}$	Coefficient matrix of domestic intermediate input used for normal exports
$A^{DP}$	Coefficient matrix of domestic intermediate input used for processing exports
$A^{MD}$	Coefficient matrix of imported intermediate input used for domestic use
$A^{MN}$	Coefficient matrix of imported intermediate input used for normal exports
$A^{MP}$	Coefficient matrix of imported intermediate input used for processing exports
$A_{v}^{D}$	Value-added coefficient matrix for domestic use
$A_{v}^{N}$	Value-added coefficient matrix for normal exports
$A_{v}^{P}$	Value-added coefficient matrix for processing exports
*X*	Total output
*M*	Total imports
$C^{M}$	Imported products used for final consumption
$I^{M}$	Imported products used for capital formation
*E^M^*	Imported products used for re-exports
TVSS	Total import value of a country’s exports per unit
$DVS^{D}$	Total domestic value-added coefficient of unit domestic consumption
$DVS^{N}$	Total domestic value-added coefficient of unit normal exports
$DVS^{P}$	Total domestic value-added coefficient of unit processing exports
TDVS	Total domestic value of a country’s exports per unit
$EEX_{domestic}$	Embodied domestic emissions in normal and processing exports
$EEX_{imported}$	Embodied imported emissions from normal and processing exports
EIM	Embodied emission in imports
BEET	Balance of embodied emission in trade
PTT	Pollution terms of trade

## Appendix A. Sector Classification

**Code**	**Sector**
1	Food products, beverages, and tobacco
2	Textiles
3	Textile products, leather, down, and footwear
4	Wood and products of wood and cork, manufacture of furniture
5	Pulp, paper, paper products, printing and publishing, stationery manufacturing
6	Coke, refined petroleum products, and nuclear fuel
7	Chemicals
8	Other non-metallic mineral products
9	Iron & steel, Non-ferrous metals
10	Fabricated metal products, except machinery and equipment
11	General and special equipment
12	Transportation equipment
13	Electrical machine and appliance
14	Communications equipment, computers, and other electronic equipment
15	Instrumentation and cultural office machinery
